# Peer review without gatekeeping

**DOI:** 10.7554/eLife.83889

**Published:** 2022-10-20

**Authors:** Michael B Eisen, Anna Akhmanova, Timothy E Behrens, Jörn Diedrichsen, Diane M Harper, Mihaela D Iordanova, Detlef Weigel, Mone Zaidi

**Affiliations:** 1 eLife Cambridge United Kingdom

**Keywords:** scientific publishing, peer review, preprints, research assessment, research communication

## Abstract

eLife is changing its editorial process to emphasize public reviews and assessments of preprints by eliminating accept/reject decisions after peer review.

Last year eLife began exclusively reviewing papers already published as preprints and asking our reviewers to write public versions of their peer reviews containing observations useful to readers (Eisen et al., 2020). Over the past 18 months we have posted eLife reviews of more than 2,200 preprints to bioRxiv and medRxiv, along with a compact editorial assessment of the significance of the findings and the strength of the evidence for them.

We have found that these public preprint reviews and assessments are far more effective than binary accept or reject decisions ever could be at conveying the thinking of our reviewers and editors, and capturing the nuanced, multidimensional, and often ambiguous nature of peer review. eLife will now let them stand on their own by publishing every paper we review, along with our reviews and an assessment as a Reviewed Preprint, a new type of research output we hope will become the norm across science.

These changes are about more than just optimizing peer review. In choosing to no longer reduce our assessments to a single, eternal publishing decision, we are relinquishing the traditional journal role of gatekeeper in favour of a new approach that restores autonomy to authors and ensures that they will be evaluated based on what, not where, they publish.

This new manifestation of eLife is available to authors immediately, and will be the only way we operate come January.

## The urgent need to fix science publishing

Peer review – the act of researchers reading, thinking carefully about, and commenting on their colleagues’ work – is an integral part of science. It is a testament to the collective spirit of the global scientific community that more than two million papers are peer reviewed every year with no compensation, and minimal recognition.

Unfortunately, much of the value generated by this immense collective effort is squandered by embedding it in a journal publishing system whose reliance on an outdated model of peer review – in which reviews are used to make accept/reject decisions and never made publicly available – strips it of most of its value.

Exposing research findings to scrutiny is an essential step in the scientific process. And asking peer reviewers to identify and help correct flaws in the authors’ methods, data and reasoning is of intrinsic value. But coupling this scrutiny to publishing decisions distorts the process, effectively turning recommendations into requirements. As a result authors often do experiments and carry out analyses that they think are unnecessary, and remove ideas and insights that they believe in from their work.

Having been written for authors and editors, the peer reviews themselves are rarely seen, their contents reduced to an accept/reject decision – a relic of pre-Internet times, when journals had to identify papers that warranted the expense of printing and mailing to subscribers. The aspects of the review that would be of most value to the community – the strengths and weaknesses of the work the reviewers identify, aspects of the findings and methods that excite them, questions that remain, how it fits in with other work and into the broader field – are all discarded once a decision is made.

Most significantly, the emphasis placed on directing papers into journals has turned journal names into the *de facto* currency of academic research careers, and institutionalized the practice of judging scientists based on where, rather than what, they publish. This has, in turn, transformed journals from a means of communicating science into gatekeepers whose judgments – ones that are heavily influenced by bias, faddishness and chance – can determine which science gets seen, and which scientists succeed.

Our choice to invest transient signals of projected quality, impact and interest with such outsized significance distorts hiring, funding and promotion decisions, corrupts research and career choices, and rewards the cynical chasing of high-impact publications over the production of genuinely impactful science that will survive the test of time. The end result is a scientific endeavour rendered less efficient, less rational, less appealing as a career and overall less effective than it should be.

In the face of these pathologies, scientists across the world are taking tangible action to make science publishing and its place in science better. They preprint their work, advocate for others to do so, and quash efforts to treat preprints as lesser works of science just because they have no journal title attached to them. They form preprint journal clubs and teach students how to write constructive comments, which they post online. They lobby colleagues and institutions to change graduation, hiring, promotion and tenure policies around publishing, and to have smart and effective open science policies. And when making their own evaluations, they choose to ignore journal titles, and to look askance at those who prioritize them.

eLife was founded with the stated mission to promote responsible behaviours in science like these. With independent funding that allows us to take steps that others fear, we are in a unique position to deliver a publishing system that scientists who care about the future of science can embrace.

## What the future of science publishing should look like

The system of science publishing we have today was not developed for today’s science or today’s technology. Its defining feature, a hierarchy of journals that use peer review to decide which papers they will publish, arose in the last century as a response to the limitations and costs of the printing press and the postal service.

In the interim, we have experienced the biggest change in the technology used to disseminate information in human history. Yet even though the Internet was literally invented to help scientists communicate with each other, it has had remarkably little impact on science communication.

We should have rebuilt this system from the ground up to take advantage of our liberation from the constraints of print. Instead, we built online submission and review systems, and replaced printed articles with pdfs, but nothing fundamental has changed. Our reliance on journal brands has stifled innovation.

We and others have long envisioned a better system, designed to serve science not publishers, and which takes full advantage of today’s technology. Its basic axioms are:

- Authors should be able to share their work freely and openly when they think it is ready.
- Peer review should consist of scientists publicly sharing their assessments of already published papers, either under the auspices of an editorial organization that oversees the review process, or on their own.
- Works of science should be reviewed by multiple relevant groups and individuals throughout their useful lifespan.

These are not new ideas: something like this was proposed by then-NIH Director Harold Varmus in 1998, but was scuttled by publisher opposition, and by the reluctance of scientists to change the system on which they had built their careers (Kling et al., 2004). But we have a real opportunity to actually do it now. All of the core elements already exist. Preprint servers like arXiv, bioRxiv and medRxiv already provide an inexpensive and universal way for authors to share their work freely and openly when they think it is ready. And eLife, Review Commons, F1000 Research, Peer Community In, PubPeer and others already publish public reviews of preprints.

## Reviewed Preprints at eLife

The essential elements of this new model – exclusively reviewing preprints and producing public peer reviews and assessments – are already core parts of the eLife editorial process. The biggest change we are making now is that we will no longer make accept/reject decisions following peer review, nor, obviously, ask our reviewers to make publishing recommendations.

The consultation between reviewers and editors that we currently use to make publishing decisions and recommendations to the authors, will now be used to craft an eLife assessment which summarizes their consensus view of the significance of findings and evaluates the strength of the evidence for them. These will be concise, written in language accessible to a non-expert reader, and will draw on a common vocabulary to ensure they are clear and consistent across the journal (see this blog post about eLife assessments for a fuller description and examples).

Although we do not have the capacity to review every preprint submitted to us, we will publish every paper we send out for review as a Reviewed Preprint, a journal-style paper containing the authors’ manuscript, the eLife assessment, and the individual public peer reviews. The authors will also be able to include a response to the reviews and assessment (see this blog post about Reviewed Preprints for examples of how they will look).

With this change in approach, we will be reducing our publication fee to $2,000, which covers all of the costs associated with overseeing peer review, publishing Reviewed Preprints and further steps described below. As always, this fee will be waived for any authors who cannot afford to pay.

We are, first and foremost, making these changes because they are good for science. And we believe the new model will be popular because it is good for scientists in all the ways we interact with the research literature. Directly associating preprints with peer reviews provides a clear value to readers, who can integrate the comments of their colleagues while they delve into the work. People who are interested in the results, but who may not be in a position to evaluate them themselves, will benefit from the perspective in eLife assessments.

Authors will benefit from a process that is simpler, has clear and certain outcomes, and which restores intellectual autonomy to them. They will have the option – but never the requirement – to submit a revised preprint that responds to those aspects of our public reviews and the private suggestions made by the reviewers that they believe merit a response. Assuming the relevant editor thinks the changes are warranted, they will send it out for rereview. Once the reviews are complete, the assessment will be rewritten and a new version of the Reviewed Preprint will be posted.

Authors are already benefitting from the recent trend towards including preprints in funding and job applications, and we hope that in most cases the committees evaluating the applications will judge the science on their own. However, since a thorough understanding of a work of science requires specialized knowledge that is not always present among the evaluators, their deliberations can benefit from concise eLife assessments and clearly written reviews from researchers with the relevant expertise.

Reviewed Preprints will receive an eLife citation and a DOI that will remain constant over versions. So, if a link to a Reviewed Preprint was included in a funding or job application, and the Reviewed Preprint is updated, the link will take readers to the latest version. There will also be ways to, where appropriate, cite and link to different versions directly.

Many databases – such as PubMed – will only index the final version of a manuscript (often called the Version of Record or VOR). Although we don’t think publishing should work this way, and hope we will evolve to a better system, we want to make sure that authors whose papers have been peer reviewed by eLife and who want them to be included in these databases have that option. Authors can, therefore, decide at any point that the latest Reviewed Preprint should become the VOR.

Before a VOR is produced, we will carry out a series of additional checks to ensure that the data, methods and code are made available, that appropriate reporting standards have been followed, that competing interest and ethics statements are complete, and that cell lines have been authenticated. As this model becomes established, we plan to carry out these checks earlier in the process and to turn these into “badges” that will appear on the Reviewed Preprint to make it easy for readers to know what established criteria the work has, and has not yet, satisfied.

It is important to note that publishing a VOR with eLife is not a requirement, and authors may wish to either let the Reviewed Preprint stand as their final version, or they may wish to retain the option to update their paper in the future.

In keeping with our commitment to author autonomy, eLife claims absolutely no control over the paper. This means that authors not only can ask a traditional journal to consider publishing their paper based on our public reviews and assessment, they are, as far as we are concerned, free to submit the paper to another organization carrying out public review, or to a traditional journal while we are reviewing it. Indeed we hope that, in the future, the norm will be for papers to be reviewed by multiple organizations with different expertise and foci at different times throughout the useful life of the paper.

## This is our chance

Nobody who interacts with the current publishing system thinks it works well, and we all recognize that the way we use it impedes scientific progress. The power to fix it resides uniquely with scientists, and we who have benefited tremendously from science, in our lives and in our careers, owe it to those whose hopes ride on its promise to not let our fear of change limit its impact one moment longer.
